# CDO-POSE: A Lightweight Model for 2D Human Pose Estimation

**DOI:** 10.3390/s26072159

**Published:** 2026-03-31

**Authors:** Haifeng Xu, Jingke Chen, Shuhan Cai, Jiangling Guo

**Affiliations:** School of Intelligent Systems Science and Engineering, Jinan University, Qianshan Street, Zhuhai 519070, China; xuhaifeng@stu.jnu.edu.cn (H.X.); jkchen@stu2024.jnu.edu.cn (J.C.); lvlvlvlvdouwa@gmail.com (S.C.)

**Keywords:** human pose estimation, YOLOv11, lightweight model

## Abstract

Human pose estimation (HPE) aims to localize human keypoints from visual inputs, which faces persistent challenges in balancing high accuracy with computational efficiency in resource constrained and real-time scenarios. To address these challenges, we propose a lightweight method named CDO-POSE based on an improved YOLOv11. Specifically, we first introduce the Context Anchor Attention (CAA) module, which is composed of three convolutional layers and two bottleneck modules to enhance feature representation while maintaining parameter efficiency. Building on this, to address the limited precision of traditional nearest-neighbor upsampling, we incorporate the Dynamic Sampling (DySample) method, which adaptively adjusts the sampling strategy according to feature importance, thereby improving upsampling accuracy. Furthermore, to align the training objective more closely with the goal of precise pose estimation, we employ the Object Keypoint Similarity Loss (OKS-Loss), which provides a more discriminative evaluation of keypoint localization errors. The experiments on MS COCO2017 and CrowdPose datasets demonstrate that our model achieves almost the same accuracy as YOLOv11s-pose with significantly fewer parameters. Moreover, the model achieves 39.79 FPS and 29.23 FPS for inference at 480p and 720p, respectively, on the NVIDIA Jetson Orin Nano, suggesting that it is suitable for real-time deployment on edge devices.

## 1. Introduction

**Figure 1:**
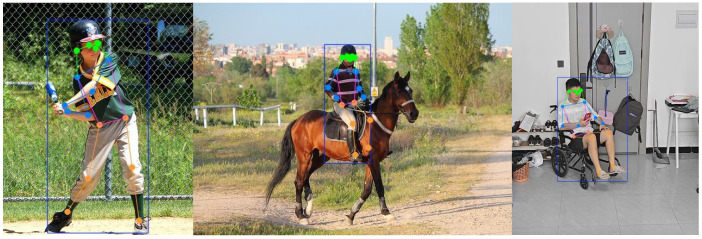
Application fields of HPE [9].

With the development of deep learning and neural networks, HPE evolves from traditional computer vision techniques to deep learning-based methods [1,2,3]. As shown in Figure 1, HPE has been widely applied in various fields. Over the years, extensive research efforts are devoted to improving the accuracy and robustness of HPE systems. These works are broadly categorized into several paradigms. The top-down methodology, pioneered by researchers such as Xiao et al. in Mask R-CNN [4], first detects individuals and then predicts their keypoints. In contrast, the bottom-up methodology, exemplified by Cao et al. in OpenPose [5], detects all body parts across the image before assembling them into individual poses. More recently, transformer-based architectures are introduced for HPE, building on the foundational work of Vaswani et al. [6], to capture long-range dependencies between joints. Similarly, graph convolutional networks, initially advanced in the context of skeleton modeling by researchers like Yan et al. [7], are effectively employed to explicitly represent the structural relationships among body parts. Together, these approaches highlight the ongoing evolution of architectural strategies aimed at overcoming the core challenges in HPE [8].

However, the pursuit of high accuracy often overlooks the critical demands of real-world applications. A significant category of these applications, such as human-computer interaction, immersive augmented reality, and real-time sports analysis, fundamentally requires real-time processing to ensure seamless and interactive user experiences [10]. Delays or high latency in such scenarios can severely degrade performance and usability. Furthermore, to achieve wide deployment and user accessibility, these applications are increasingly expected to run on edge devices, including mobile phones, embedded systems, and UAVs [11]. To et al. [12] implemented a 3D human pose estimation model on the Google Coral Dev Board, which integrates an NXP i.MX 8M SoC with a quad-core ARM Cortex-A53 CPU and a Google Edge TPU delivering up to 4 TOPS of AI performance at around 2 W power, equipped with 1 GB LPDDR4 memory and 8 GB eMMC storage. Liu et al. [13] and Zhang et al. [14] deployed their human pose estimation frameworks based on YOLO and AlphaPose, and BlazePose with ST-GCN, on the NVIDIA Jetson Nano platform, which features a quad-core ARM Cortex-A57 CPU and a 128-core Maxwell GPU, includes 4 GB of LPDDR4 memory, and operates within a 5–10 W power envelope, achieving promising real-time performance and stable inference on edge devices. These studies collectively indicate that with appropriate model optimization, accurate and efficient human pose estimation can be achieved even on low-cost edge devices. The use of such hardware is driven by compelling needs for low power consumption, small form factors, cost-effectiveness, and data privacy, which are hallmarks of edge computing paradigms [15]. Many high-accuracy models are too computationally intensive for real-time inference, particularly in multi-person video scenarios [16]. This is especially evident in top-down pose estimation frameworks such as AlphaPose [17], which require running a separate pose estimation network for each person detected in a frame. This sequential processing paradigm leads to a computational cost that scales linearly with the number of people, resulting in high latency and making it unsuitable for real-time applications on standard hardware. This computational burden is especially challenging on edge devices, where limited memory, power, and processing capacity are well-documented constraints [18]. Although methods like OpenPose [5] have achieved near real-time performance on mid-range GPUs, they still face a significant trade-off between accuracy and efficiency when deployed on commercial edge hardware, as shown in comparative studies [19]. Consequently, there remains a critical need for more efficient HPE methods that can deliver satisfactory performance without excessive resource requirements, enabling better interactive experiences in practical applications.

**Figure 2:**
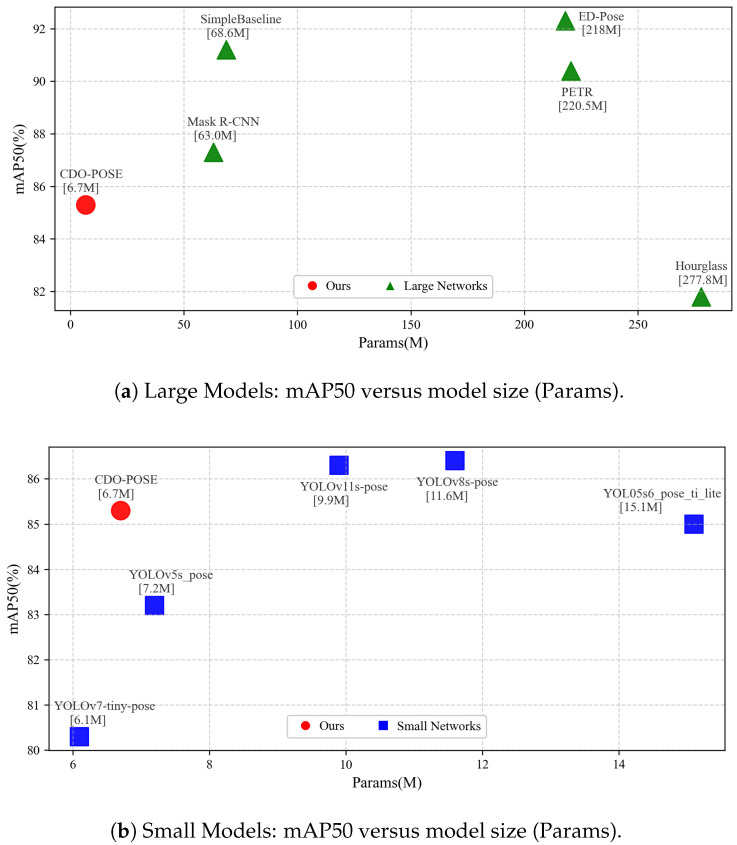
Scatterplot of model performance versus parameter count on COCO 2017.

Despite recent progress, edge deployment still suffers from a dual gap: backbone simplification often harms fine-grained keypoint cues, whereas high-accuracy models remain too heavy for real-time embedded inference. We therefore adopt YOLOv11 as a strong single-stage baseline and modify it at the backbone, feature-fusion, and loss levels to better balance compactness and localization accuracy. As illustrated in Figure 2, our model achieves remarkable performance while maintaining a significant advantage in parameter efficiency.

To minimize hardware costs, this paper proposes a lightweight model for real-time 2D multi-person HPE. The key contributions of this work include:

- We introduce an improved C3k2CAA module, an attention-augmented structure, to replace the original structure in the backbone network, achieving a reduction of 32.3% parameters while maintaining accuracy.
- Employing DySample as upsampling instead of conventional interpolation increases accuracy without increasing model complexity.
- Incorporating the OKS-Loss function further boosts the accuracy of the estimation without increasing the number of parameters.

## 2. Related Work

**Figure 3:**
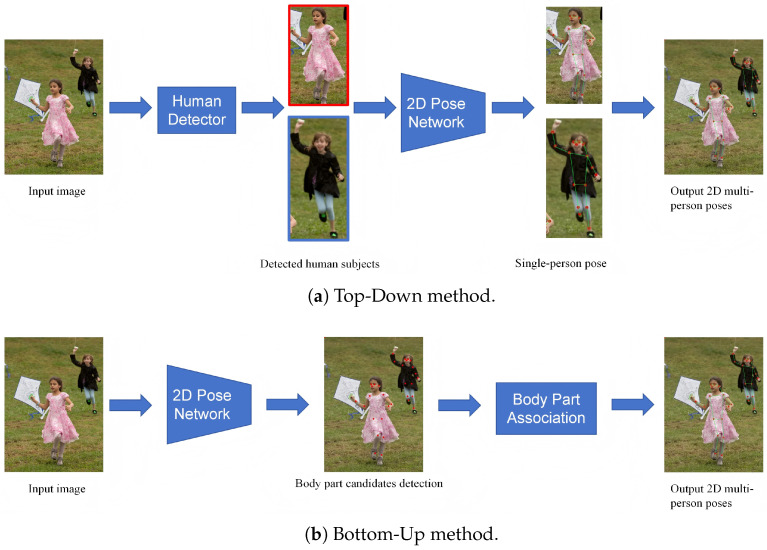
Illustration of the multi-person 2D HPE frameworks: two methods for HPE, top-down and bottom-up [9].

Mainstream multi-person 2D HPE methods include top-down and bottom-up approaches, as shown in Figure 3, which illustrates the general framework for multi-person pose estimation.

Top-down methods first locate all human bounding boxes in an image through object detection, and then perform keypoint detection for each detected human region to predict the positions of the keypoints [20,21,22,23,24]. Papandreou et al. [20] proposed a two-stage architecture for keypoint prediction, which performs well in occluded and cluttered scenes. However, its two-stage process (detection and keypoint prediction) leads to slower inference speed, especially under high-resolution inputs or in multi-person scenarios. Fang et al. [21] designed a Regional Multi-person Pose Estimation (RMPE) algorithm to improve HPE performance in complex scenes. However, their proposed symmetric SSTN and parallel SPPE increase the model’s complexity. Yuan et al. [24] combined HRNet with Transformer to propose HRFormer, which significantly improves pose estimation accuracy by maintaining high-resolution feature flows and fusing multi-scale information. However, when handling extremely high-resolution inputs, the multi-resolution parallel design may still introduce substantial memory and computational overhead.

Bottom-up methods directly predict all human keypoints across the entire image, and then use clustering or association algorithms to group the keypoints into different human instances, forming the final human skeleton [5,25,26,27,28,29,30,31]. Kreiss et al. [25] proposed a method called PifPaf, which performs excellently in low-resolution and dense occlusion scenarios. However, its ResNet-based backbone requires high GPU computing power. Newell et al. [26] proposed a method that outputs detection heatmaps and embedding labels simultaneously through a single-stage network, avoiding the complex post-processing of traditional multi-stage workflows. While this simplifies the process and improves efficiency, the method’s reliance on stacked hourglass networks and 512 × 512 inputs results in high training and inference computational overhead. Geng et al. [28] proposed the DEKR algorithm, which uses decoupled representation learning and multi-branch structures to significantly improve localization accuracy.

Overall, the aforementioned methods all involve high computational complexity and a large number of parameters, making them difficult to deploy on edge devices.

In recent years, there has been a growing focus on enhancing the real-time performance and lightweight nature of HPE models. OpenPose demonstrates real-time inference capability on powerful GPUs in multi-person settings [5]. However, its high parameter count and computational complexity still restrict its applicability on edge devices. In absolute terms, OpenPose remains heavy (e.g., VGG-type backbone with multi-stage refinement), implying sizable parameters and FLOPs. Thus it is efficient but not lightweight, and deployment on edge devices remains challenging [5,16]. To address this, several studies have pursued further lightweight solutions. For example, lightweight OpenPose adopts MobileNet as the backbone to significantly reduce both parameters and computations, enabling real-time inference on CPUs [16]. Kang et al. [32] proposes an angle-based action recognition method that uses the cosine theorem to analyze joint angles for quick behavior without relying on complex networks. While these approaches achieve notable improvements in speed, they often come at the cost of reduced accuracy, particularly in challenging scenarios such as occlusions, complex poses, or multi-person interactions [33,34]. More recent lightweight HPE studies have therefore paid increasing attention to preserving spatial representations and improving computational efficiency simultaneously. Representative efforts include lightweight high-resolution or transformer-based designs, such as PoseSynViT [35] as well as more adaptive and efficient estimation frameworks, including DynPose [36] and LGM-Pose [37]. Although these methods further improve the practicality of real-time HPE, maintaining precise keypoint localization under strict computational constraints remains a challenging problem.

To achieve high accuracy and generalization capability in HPE, the number of parameters in the model inevitably increases, which poses significant challenges for real-time performance. An alternative research direction, however, focuses on optimizing this trade-off, striving to reduce the model’s size while preserving accuracy as much as possible. This paper explores this perspective, aiming to identify an optimal balance between model complexity and inference speed. By doing so, we enhance the real-time performance of HPE and improve its feasibility for deployment on edge devices.

## 3. Methodologies

### 3.1. Preliminary

YOLOv11, as the latest model introduced by Ultralytics, offers significant advancements in computer vision. In the backbone network of YOLOv8, the C2f module enhances feature map connectivity by splitting the feature maps into multiple parts, processing them separately, and then merging them [38]. However, this design can lead to computational redundancy, increasing both computational cost and the number of parameters. To address this, YOLOv11 introduces a more efficient C3k2 block, which is based on the Cross-Stage Partial network. The C3k2 block employs two smaller convolutions to reduce computational cost while maintaining model performance [39]. In YOLOv8, there are limitations in effectively capturing spatial dependencies and focusing on regions of interest in scenarios involving small, occluded, or overlapping objects. To address these issues, YOLOv11 integrates the Context-Aware Position-Sensitive Attention (C2PSA) mechanism [40], which effectively focuses on regions of interest, improving detection accuracy for objects with varying sizes and positions.

**Figure 4:**
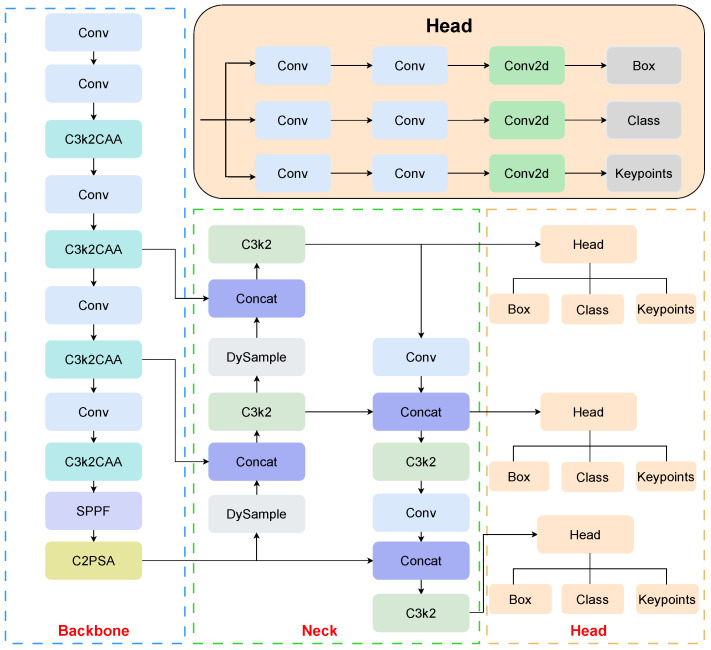
Architecture of the proposed CDO-POSE network, featuring a backbone equipped with successive C3k2CAA blocks and the DySample upsampler for enhanced feature extraction.

However, when deployed on edge devices such as the Jetson Orin Nano, YOLOv11’s large parameter size presents significant challenges in terms of efficiency and performance. In response to this limitation, as illustrated in Figure 4, we propose an improved version based on YOLOv11. Specifically, the original C3k2 modules are replaced with more efficient C3k2CAA modules in the backbone to reduce parameter redundancy while preserving contextual representation capability. Since backbone compression may weaken fine-grained spatial details, the original upsampling operators in the neck are further replaced with the DySample module to enhance feature fusion and recover detailed localization information. In addition, OKS-Loss is introduced into the keypoint branch during training to provide scale-aware supervision for pose estimation. In this way, C3k2CAA, DySample, and OKS-Loss collaboratively improve representation efficiency, feature reconstruction, and optimization supervision, respectively, making the overall model better suited for edge deployment while balancing parameter efficiency and detection performance.

In the final pipeline, all C3k2 blocks in the backbone are replaced by C3k2CAA, while the stage interfaces and the overall FPN topology remain unchanged to preserve the original multi-scale feature transmission path. Meanwhile, the nearest-neighbor upsampling operators in the neck are replaced by DySample, enabling adaptive feature reconstruction during the top-down fusion process. During training, the box regression and classification branches remain unchanged, whereas only the keypoint branch is optimized with OKS-Loss. This integration makes the role of each module explicit and keeps their contributions within the complete pipeline easy to analyze.

### 3.2. C3k2 with Context Anchor Attention

**Figure 5:**
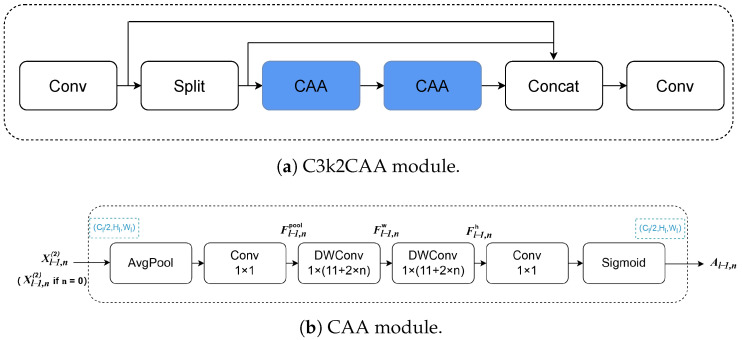
Structure of C3k2CAA module.

In the YOLOv11 backbone, the original C3k2 module utilizes C3k and Bottleneck blocks for feature extraction [41]. However, these blocks contain multiple standard convolutional layers, where the number of parameters increases with both the kernel size and the number of channels, resulting in quadratic growth and a high parameter count. Cai et al. [42] demonstrate that combining multi-scale convolution kernels with the Context Anchor Attention (CAA) mechanism effectively captures both local and long-range contextual information while maintaining lightweight efficiency. To achieve lightweight efficiency without sacrificing accuracy, we redesign the YOLOv11 backbone by replacing the original C3k2 modules with the improved C3k2CAA. The C3k2CAA module depicted in Figure 5a, integrates the Context Anchor Attention (CAA) mechanism described in Figure 5b [42]. In our redesign, the original C3k and Bottleneck blocks are replaced with the CAA module, which is itself built upon the works of [41,43,44]. CAA aims to capture contextual interdependencies among distant pixels while simultaneously enhancing central features. As illustrated in Figure 5b, within the *n*-th PKI Block of the *l*-th stage, CAA adopts average pooling followed by a 1×1 convolution to obtain the local region feature:

(1) $$F_{l-1,n}^{\mathrm{pool}}={\mathrm{Conv}}_{1\times 1}\;(P_{\mathrm{avg}}\;(X_{l-1,n}^{\left(2\right)})),\;n=0,\ldots ,N_{l}-1$$

where $P_{\mathrm{avg}}$ represents the average pooling operation and for n=0, $X_{l-1,n}^{\left(2\right)}=X_{l-1}^{\left(2\right)}$. To approximate a standard large-kernel depth-wise convolution, CAA applies two depth-wise strip convolutions:

(2) $$\begin{array}{cc} F_{l-1,n}^{w} & ={\mathrm{DWConv}}_{1\times k_{b}}\;\left(F_{l-1,n}^{\mathrm{pool}}\right)\\ F_{l-1,n}^{h} & ={\mathrm{DWConv}}_{k_{b}\times 1}\;\left(F_{l-1,n}^{w}\right) \end{array}$$

In the original design of CAA, this design choice is motivated by two key advantages: first, depth-wise strip convolution is lightweight, as a similar effect to a conventional $k_{b}\times k_{b}$ 2D depth-wise convolution is achieved with a pair of 1D depth-wise kernels, reducing parameters by approximately $k_{b}/2$; second, depth-wise strip convolution is effective in identifying and extracting features of objects with slender shapes, such as human pose. To increase the receptive field of CAA Module as the network goes deeper, the kernel size is set as $k_{b}=11+2\times l$, i.e., the kernel size $k_{b}$ is calculated as a function of the stage depth *l*. Such a design enhances the ability of CAA to establish the relationship between long-range pixels, while keeping the computational cost under control owing to the depth-wise strip depth-wise design, which is consistent with our objective of achieving a lightweight architecture.

### 3.3. DySample

YOLOv11 uses nearest neighbor interpolation for upsampling, which replicates the values of the nearest pixels to restore feature map resolution. However, nearest neighbor and bilinear interpolations only consider sub-pixel neighborhood, failing to capture the rich semantic information required by dense prediction tasks [45]. Moreover, if the input image contains noise, the noise is amplified during upsampling, thereby affecting the prediction accuracy. To address these issues, we adopt DySample, a lightweight and efficient dynamic upsampling method, to replace the traditional nearest neighbor interpolation used in the original upsampling process [46].

**Figure 6:**
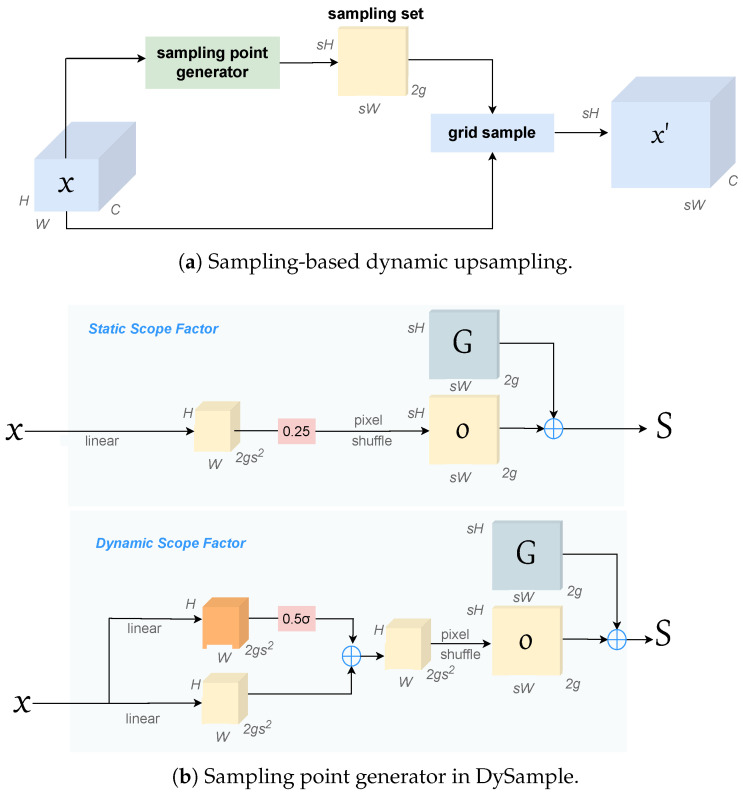
Sampling point generator in DySample.

DySample bypasses dynamic convolutions and implements upsampling from a point sampling perspective. It does not require high-resolution images and efficiently performs feature resampling using learned dynamic offsets. The design of DySample is illustrated in Figure 6.

The workflow of DySample begins with the input feature map $X\in R^{C\times H\times W}$ and an upsampling scale factor *s*. DySample first projects the input feature map *X* into an offset tensor *O*, which captures the spatial shifts needed for resampling. The size of the offset tensor *O* is $2gs^{2}\times H\times W$, where *s* is the upsampling scale factor and *g* denotes the number of channel groups used to organize offsets. The generated offset *O* is then adjusted to match the final upsampling size. This process is represented by the following equation:

(3) O=Linear(X)

Next, the offset *O* is reshaped using a pixel rearrangement algorithm to a new size of 2g×sH×sW, forming the new sampling set *S*. The sampling set *S* is obtained by adding the original sampling grid *G* to the offset *O*, ensuring precise spatial mapping during the upsampling process. This is given by:

(4) S=G+O

The generated sampling set *S* is used with the grid sample function to resample the input feature map *X*, generating the upsampled feature map $X^{\prime }$ with dimensions C×sH×sW. This resampling process ensures that the resulting feature map maintains high resolution and effectively preserves important details while reducing noise. The process is described by:

(5) $$X^{\prime }=\mathrm{grid}\;\mathrm{sample}\left(X,S\right)$$

The sampling point generator is instantiated in two different ways, as illustrated in Figure 6b. The first variant adopts a static scope factor, where the predicted offsets are uniformly scaled by a fixed constant (0.25) before being added to the base sampling grid. This provides a simple and efficient way to constrain the offset magnitude and stabilize training. The second variant introduces a dynamic scope factor, in which an additional lightweight 1×1 convolution predicts a scope map. After applying a sigmoid activation and a scaling factor of 0.5, the scope map adaptively modulates the offsets according to the local content. This design allows the network to adjust the offset magnitude in a data-dependent manner, enabling more flexible and content-aware resampling. Since the Dynamic Scope Factor introduces additional computational overhead, we adopt the Static Scope Factor in our implementation. This design reduces the magnitude of the offsets, which helps mitigate noise amplification during the upsampling process. By constraining the range of offsets, we ensure that the model does not amplify unwanted distortions, thus maintaining the accuracy of keypoint predictions. The use of the Static Scope Factor stabilizes the loss function and helps preserve the model’s performance, ensuring that accuracy is maintained without introducing significant computational complexity.

Furthermore, DySample does not require customized CUDA packages, as it can be efficiently implemented with standard built-in functions in PyTorch 2.1.1. Moreover, DySample achieves higher model accuracy without introducing additional parameters or computational overhead.

### 3.4. OKS-Loss

The original keypoint loss function in YOLOv11, which computes the squared L2 distance between predicted and ground truth keypoints, is sensitive to outliers and large errors, often leading to unstable gradient behavior during training [38]. Furthermore, the loss normalization strategy, which calculates a keypoint loss factor based primarily on visible keypoints, leads to imbalanced gradient contributions in cases of highly occluded or sparsely annotated poses. This approach lacks explicit mechanisms to adapt to the varying perceptual importance of different keypoints, thereby limiting its ability to achieve fine-grained localization accuracy required for robust pose estimation. To address these issues, we replace the original keypoint loss function with the Object Keypoint Similarity (OKS) loss proposed in YOLO-Pose [47]. The OKS-Loss directly regresses keypoints relative to anchor centers, optimizing the evaluation metric itself. It is inherently scale-invariant and assigns higher penalties to errors in semantically critical keypoints (e.g., eyes, nose, ears) compared to less critical ones (e.g., shoulders, knees). The OKS-Loss is formulated as:

(6) $$L_{\mathrm{kpts}}=1-\frac{\sum_{n=1}^{N_{\mathrm{kpts}}}\exp\;\left(-d_{n}/\left(2s^{2}k_{n}^{2}\right)\right)\;\delta \left(v_{n}>0\right)}{\sum_{n=1}^{N_{\mathrm{kpts}}}\delta \left(v_{n}>0\right)}$$

where $d_{n}$ represents the distance between the predicted and ground truth for the *n*th keypoint, $k_{n}$ is the keypoint specific weight, and *s* denotes the object scale. $\delta (v_{n}>0)$ acts as the visibility flag for each keypoint.

(7) $$d_{n}^{L1}=|x_{\mathrm{pred},n}-x_{\mathrm{gt},n}\left|+\right|y_{\mathrm{pred},n}-y_{\mathrm{gt},n}|$$

YOLOv11 uses L2 distance, while OKS-Loss adopts L1 distance (Equation (7)). The L1 loss has relatively constant gradients in the case of large errors, which reduces the risk of gradient explosion. Additionally, L1 does not excessively penalize small errors, allowing the model to maintain a stable learning process even in precise localization tasks.

YOLOv11 normalizes loss per visible keypoint, whereas OKS-Loss includes both visible and invisible points (Equation (8)):

(8) $$\lambda =\frac{N_{\mathrm{total}}+N_{\mathrm{inv}}}{\sum_{n=1}^{N_{\mathrm{total}}}\delta \left(v_{n}>0\right)+\epsilon }$$

The original YOLOv11 loss factor normalizes the loss based on the number of visible keypoints per sample, ensuring that the loss is averaged only over the visible keypoints. When the number of visible keypoints is small, the weight of each individual keypoint is increased. On the other hand, OKS-Loss includes both visible and invisible keypoints in the numerator (Equation (8)), altering the treatment of invisible or invalid points. This adjustment incorporates invisible keypoints (or invalid points) into the total count, ensuring that the proportion of keypoint loss within the total loss remains more stable in multi-object and multi-person scenarios. This further prevents an imbalance caused by an over-amplification of the loss when only a few visible keypoints are present in some samples.

OKS-Loss introduces a hybrid loss combining the OKS exponential term and L1 regularization (Equation (9)):

(9) $$\begin{array}{cc} L_{\mathrm{hybrid}} & =\lambda \cdot \left(1/N_{\mathrm{vis}}\right)\sum_{n=1}^{N_{\mathrm{kpts}}}\left[\alpha \cdot \left(1-\exp\;\left(-d_{n}/\left(2k_{n}^{2}s\right)\right)\right)+\beta \cdot d_{\mathrm{abs},n}\right]\cdot m_{n} \end{array}$$

where α=0.1 and β=0.9 are empirically chosen coefficients, λ is the loss factor defined in Equation (8), $m_{n}$ is a binary mask indicating whether the keypoint should be included in the loss calculation. The exponential term provides fine-grained control for small deviations, while the L1 term ensures stable gradients for large deviations, thereby enhancing the model’s ability to correct outliers and improving overall convergence.

## 4. Experiments

### 4.1. Datasets

In this study, we select the MS COCO2017 and CrowdPose datasets to comprehensively evaluate the performance of the proposed CDO-POSE method across different scenarios. These two datasets are chosen not only because they are widely recognized benchmarks, but also because they meet the practical requirements of our target application on edge devices. They both contain the necessary annotation information, including human bounding boxes and keypoints, which ensures that the training and evaluation of our model are fully supervised. In addition, they feature a large number of crowded scenes with frequent occlusions, which resemble real deployment environments where multiple people often appear in a single frame. Moreover, the image resolutions of these datasets are compatible with common edge devices’ inputs, allowing for a balanced evaluation of accuracy and computational cost.

The MS COCO2017 dataset, as one of the most influential benchmark platforms in the field of computer vision, is widely used for performance evaluation and comparative analysis of HPE algorithms [9]. The dataset is divided into train, validation, and test sets, containing more than 200,000 images and 250,000 people. Each annotation instance is marked using a dual annotation mechanism: for object detection, the spatial position of the human body in the scene is precisely marked using rectangular or polygonal bounding boxes; for pose estimation, the human skeletal structure is constructed through 17 anatomical keypoints, including head joints, limb endpoints, and torso core nodes, while also incorporating challenging real-world situations such as occlusions and truncations. The diversity and complexity of the MS COCO2017 dataset make it an ideal choice for evaluating pose estimation algorithms in real-world conditions, providing a comprehensive assessment of the CDO-POSE method across varied scenarios. MS COCO2017 dataset provides both bounding box and keypoint annotations while containing many images with multiple people and partial occlusions, which closely resemble edge devices’ application scenes. We evaluate our method using resized inputs that align with common devices’ resolutions, ensuring that the results reflect the actual performance-efficiency trade-offs in edge deployment.

The CrowdPose dataset is a large-scale benchmark specifically designed for multi-person pose estimation in crowded scenes [48]. It consists of 20,000 images densely populated with approximately 80,000 annotated person instances, capturing a wide range of real-world crowd activities and scenarios from various viewpoints and distances. This dataset stands out for its large scale, diversity, and high crowd density, often including complex occlusions and overlaps. Each person in the dataset is annotated with 14 body keypoints, which are manually verified for accuracy. The dataset is divided into three subsets: 10,000 images for training, 2000 images for validation, and 8000 images for testing. The unique challenges posed by the high crowd density and the frequent occlusions between people make the CrowdPose dataset an essential tool for testing the accuracy of our method in scenarios with overlapping poses. Compared with MS COCO2017 dataset, the CrowdPose dataset emphasizes denser and more complex interactions among people, providing a more demanding test for algorithms that must handle overlapping poses and occluded limbs. It also offers high-quality bounding box and keypoint annotations, while the image scale remains consistent with typical edge devices’ resolutions. This makes CrowdPose dataset a suitable complement to MS COCO2017 dataset, for verifying the robustness and adaptability of the proposed CDO-POSE method under practical, crowded conditions.

### 4.2. Baselines

We respectively compare large and small models to highlight their representative design choices and performance differences. For the large-model baselines, we compare the following baselines:

- Stacked Hourglass network employs repeated encoder-decoder hourglass modules to capture multi-scale context and iteratively refine heatmaps for precise keypoint localization [49].
- PETR is a transformer-based end-to-end pose estimator that casts multi-person pose as a DETR-style set prediction problem and uses learned queries to directly output human keypoints without separate detection or grouping [50].
- ED-Pose unifies person detection and pose estimation by treating keypoints as explicit detection targets and decoding them with queries so that a single network handles multi-person pose in a fully end-to-end manner [51].
- SimpleBaseline follows a top-down design with a ResNet backbone and a small stack of transposed convolutions to produce heatmaps, showing that a minimal architecture is highly competitive [34].
- Mask R-CNN extends a two-stage detector with a dedicated keypoint branch that predicts human joints together with bounding boxes and segmentation masks, enabling joint detection and pose within one framework [4].

For the small-model baselines, we compare the following lightweight YOLO-based pose estimators:

- YOLO5s6_pose_ti_lite modifies YOLOv5 to directly regress body joints in a single forward pass and couples person detection with keypoint regression for low-latency inference [47].
- YOLOv8s-pose adds a dedicated keypoint head on top of a lightweight YOLOv8 backbone and uses decoupled detection heads to deliver strong accuracy at real-time speeds [38].
- YOLOv11s-pose further refines the YOLO design with an improved backbone and head to raise pose accuracy while preserving throughput [52].
- YOLOv5s_pose adapts YOLOv5 to output keypoint coordinates for each detected person and provides a simple and fast baseline [47].
- YOLOv7-tiny-pose is an extremely compact variant that emphasizes high-speed inference with a lightweight pose head and accepts some accuracy trade-offs [53].

### 4.3. Training Details

All experiments in this paper are conducted on the same platform, equipped with a 12th Gen Intel i7-12700 CPU, a single NVIDIA GeForce RTX 4090 D GPU with 24 GB of VRAM, and CUDA 12.5. The input image size is set to 640 × 640. In general, larger input resolutions can provide richer spatial information and potentially improve model accuracy. However, higher resolutions also significantly increase computational cost and training time. Preliminary experiments show that training with higher resolutions (e.g., 960 × 960) would dramatically increase the training time on our platform, requiring approximately four times the training time compared to the 640 × 640 setting, making the overall training process extremely time-consuming. Therefore, 640 × 640 is selected as a practical trade-off between training efficiency and detection performance. The data augmentation strategies applied include random image translation with a probability of 0.1, random scaling with a probability of 0.5, and mosaic augmentation with a probability of 1.0. Unless otherwise stated, all ablation experiments and lightweight YOLO-style comparisons are conducted using the same input resolution, optimizer, data augmentation strategy, and evaluation protocol to ensure fair comparison. The model is trained using the SGD optimizer with a weight decay of 0.0005, an initial learning rate of 0.01, which is reduced to 0.0001 using cosine annealing [54]. The training is performed separately on the COCO2017 and CrowdPose datasets for up to 1500 epochs, where the relatively long schedule is adopted to promote stable convergence under strong data augmentation. To prevent overfitting, an early stopping mechanism is implemented, halting the training if no significant improvement in mAP is observed over 100 consecutive iterations. These hyperparameters were selected based on common practices in related studies and validation performance. During the training stage, we observed that the model maintained stable performance within reasonable ranges of these hyperparameters, suggesting that the proposed method is not highly sensitive to moderate hyperparameter variations.

### 4.4. Experimental Results

**Figure 7:**
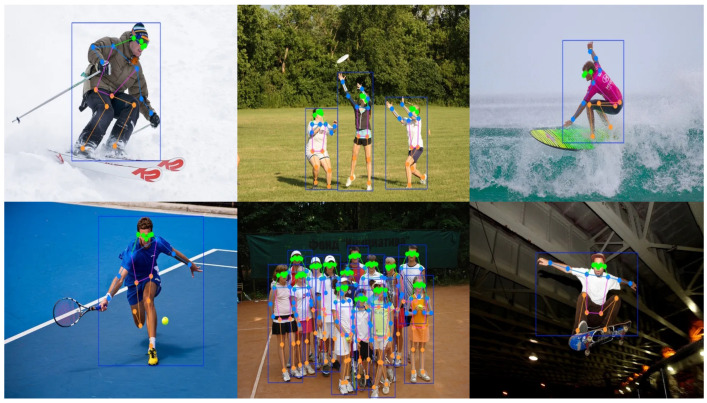
Visualization results for CDO-POSE [9].

On the COCO2017 test-dev benchmark, CDO-POSE achieves an mAP@50 of 85.3 with only 6.7 M parameters, and on the CrowdPose dataset it reaches an mAP@50 of 84.6 with the same parameter count, as reported in Table 1 and Table 2. Rather than claiming universally superior performance over all heavier architectures, these results suggest that CDO-POSE occupies a favorable position on the overall accuracy-efficiency spectrum. Moreover, when compared with the lightweight YOLO family, which represents a more deployment-relevant single-stage baseline, CDO-POSE remains competitive in accuracy while requiring a markedly smaller parameter budget across both datasets. It should be noted that, for inter-model comparison, we emphasize parameter count and benchmark accuracy for inter-model comparison. Although GFLOPs can reflect arithmetic complexity, the compared methods in this study differ substantially in architecture and inference pipeline, including top-down, bottom-up, transformer-based, and one-stage YOLO-based designs. Since GFLOPs reported in the literature are often obtained under different input resolutions and counting protocols, directly comparing these values may introduce unfairness. Therefore, we use parameter count as a unified compactness indicator in the benchmark comparison. As illustrated in Figure 7, the visualization results of the model inference are presented.

For the large-model group, on the COCO2017 test-dev, CDO-POSE uses 6.7 M parameters and reaches mAP@50 of 85.3, surpassing Hourglass at 277.8 M and mAP@50 of 81.8 by 3.5 points, while also improving mAP@75 by 0.3 and mAP@50-95 by 0.4, which corresponds to 97.6% fewer parameters. Relative to ED-Pose at 218 M and mAP@50 of 92.3, CDO-POSE uses 96.9% fewer parameters with a 7.0-point gap in mAP@50. On CrowdPose, CDO-POSE uses 6.7 M parameters and exceeds Mask R-CNN at 63.0 M by 1.1 points in mAP@50 while cutting parameters by 89.5 percent, and compared with ED-Pose at 218 M and mAP@50 of 90.5 it reduces parameters by 97.0% with a 5.9-point gap in mAP@50. These results indicate that the proposed architectural choices effectively balance compactness and accuracy.

For the small-model group, on the COCO2017 test-dev dataset, CDO-POSE outperforms YOLOv7-tiny-pose at 6.1 M by 5.0 points on mAP50, from 80.3 to 85.3, and by 14.8 points on mAP75, from 47.3 to 62.1, reflecting stronger high-threshold localization. Relative to YOLOv8s-pose at 11.6 M, CDO-POSE uses 42.2% fewer parameters while trailing by only 1.1 points on mAP50 and 1.7 points on mAP50-95, which leads to a better accuracy-efficiency trade-off. The same trend holds on CrowdPose, where CDO-POSE surpasses YOLOv7-tiny-pose by 5.9 points at mAP50, by 12.3 points at mAP75, and by 18.4 points on mAP50-95. These results demonstrate that CDO-POSE achieves an excellent balance between model size and accuracy, making it well-suited for deployment in edge devices.

### 4.5. Ablation Study

**Figure 8:**
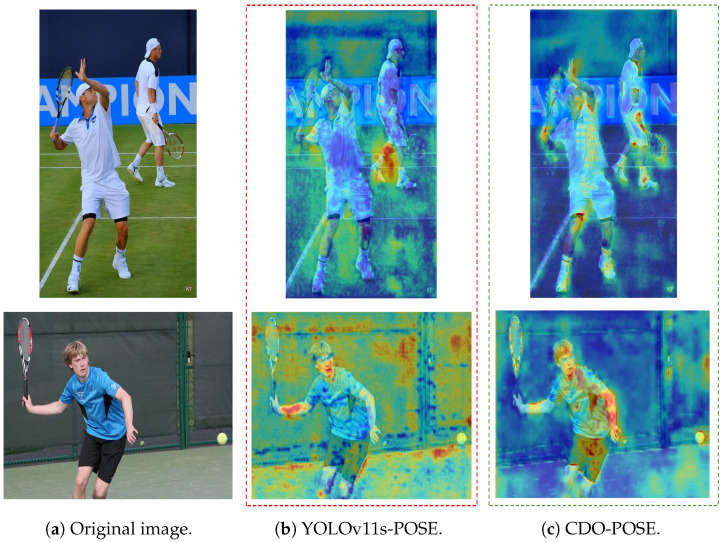
Heatmap visualization of the baseline (YOLOv11s-POSE) vs. CDO-POSE [9].

To evaluate the impact of each individual improvement and their combinations on model performance, we conduct ablation experiments on the COCO2017 test-dev dataset. As shown in Table 3, the baseline YOLOv11s-pose achieves 57.3 mAP@50-95 with 9.92 M parameters [52]. When integrating the C3k2CAA module alone, the parameter count reduces by 32.3% to 6.7 M, while mAP@50-95 slightly drops by 2.4 points to 54.9, indicating that C3k2CAA is primarily a compression-oriented module rather than a purely accuracy-driven one. In this sense, its value should be understood from the perspective of deployment trade-off: although used alone it introduces a 2.4-point drop in mAP@50-95, it substantially improves model compactness, which is consistent with the edge-side speedup observed in Section 4.6. Therefore, the role of C3k2CAA is not to maximize accuracy at any computational cost, but to provide a more favorable operating point for efficient deployment. C3k2CAA effectively balances efficiency and feature representation, where the reduction in parameters does not lead to a significant loss of accuracy. Introducing DySample alone increases mAP@50-95 by 0.4 points to 57.7, demonstrating that the module improves performance without introducing additional parameters. This improvement is attributed to DySample’s ability to enhance edge-preserving upsampling and thus spatial precision, recovering finer details. Notably, OKS-Loss provides the most significant accuracy boost, increasing mAP@50-95 by 2.5 points to 59.8 without adding any parameters, which demonstrates that the OKS-Loss function better fits human keypoint distributions during training, thereby improving model accuracy. When C3k2CAA is combined with the two accuracy-oriented modules DySample and OKS-Loss, it largely compensates for the compression-induced drop, yielding 57.0 mAP@50-95 at 6.7 M on COCO2017 and enabling the model to maintain a stronger focus on the human torso region, which supports robust reasoning under occlusion and explains the gains on CrowdPose. More importantly, the final model improves mAPL from 65.7 to 67.1, suggesting that the proposed design achieves a better balance between efficiency and accuracy. We employ Grad-CAM++ to visualize the heatmap results shown in Figure 8 [55], the visualization indicates that CDO-POSE better focuses on the human torso region, achieving more effective pose estimation.

It is worth noting that we find the DySample and OKS-Loss configuration added no additional parameters and increased mAP@50-95 by 2.6%. This offers an excellent option for scenarios that require higher accuracy without the need for increased model complexity. From an efficiency perspective, however, C3k2CAA is the only modification that directly reduces model size and memory footprint, making it the main source of acceleration on edge devices. By comparison, DySample introduces no additional learnable parameters and only limited operator-level overhead in our implementation, whereas OKS-Loss is used only during training and therefore has no effect on inference latency.

### 4.6. Edge Device Deployment

To validate the practical deployment capabilities of CDO-POSE, we conduct various evaluations on the NVIDIA Jetson Orin Nano edge computing platform. Considering that edge devices often process video streams with different resolutions in real-world scenarios, we evaluate the inference performance under commonly used video resolutions such as 480p and 720p. The Jetson Orin Nano features an Ampere-architecture GPU with 128 cores and a 6-core ARM Cortex-A78AE CPU, delivering up to 67 TOPS of AI computing performance while consuming only 10–15 watts, and is equipped with 8 GB of memory. This device represents a typical embedded system used in real-world applications such as mobile robots, drones, and smart surveillance systems.

**Figure 9:**
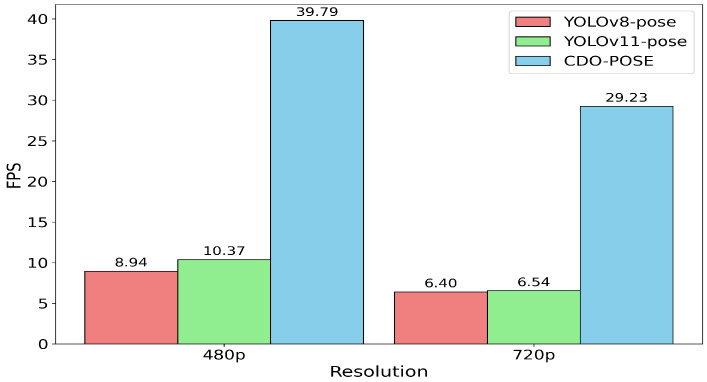
Visualization results on Jetson Orin Nano.

To evaluate the inference performance, we use an 82-s video featuring six people performing a dance, in two versions: 908 × 480 (480p) at 30 FPS and 1364 × 720 (720p) at 30 FPS. The video includes challenging elements such as movement, occlusion, camera tilt, and stretching. Table 4 reports the FPS of the network inference stage on the Jetson Orin Nano, where video decoding and post-processing are not included, and the visualization results of time are shown in Figure 9. The model achieves 39.79 FPS at 480p resolution and 29.23 FPS at 720p resolution. In comparison, YOLOv8-pose with a parameter size of 69.4 M achieves only 8.94 FPS at 480p resolution and 6.40 FPS at 720p resolution, suffering from a drastic drop in inference speed and failing to meet real-time requirements on edge devices. These FPS results provide a more direct evaluation of practical deployment efficiency on edge hardware than theoretical complexity metrics alone, because they reflect the combined effect of model structure, memory access, and hardware execution.

Furthermore, to ensure consistent accuracy across different hardware environments, we evaluate CDO-POSE on PC-grade GPUs and edge devices using the COCO2017 validation and test datasets. As shown in Table 5, the results from both the COCO2017 validation and test sets show that CDO-POSE’s performance on the Jetson Orin Nano is close to its performance on the high-performance NVIDIA 4090D GPU. This performance demonstrates the efficiency of CDO-POSE on edge devices, while significantly reducing computational overhead without sacrificing accuracy, making it suitable for real-time applications on edge devices. In this part, GFLOPs are reported only for CDO-POSE as a reference to describe the computational cost of the proposed model under the unified experimental setting, while the efficiency comparison among different models is mainly based on Params in Table 1 and Table 2 and FPS in Table 4.

### 4.7. Limitations

**Figure 10:**
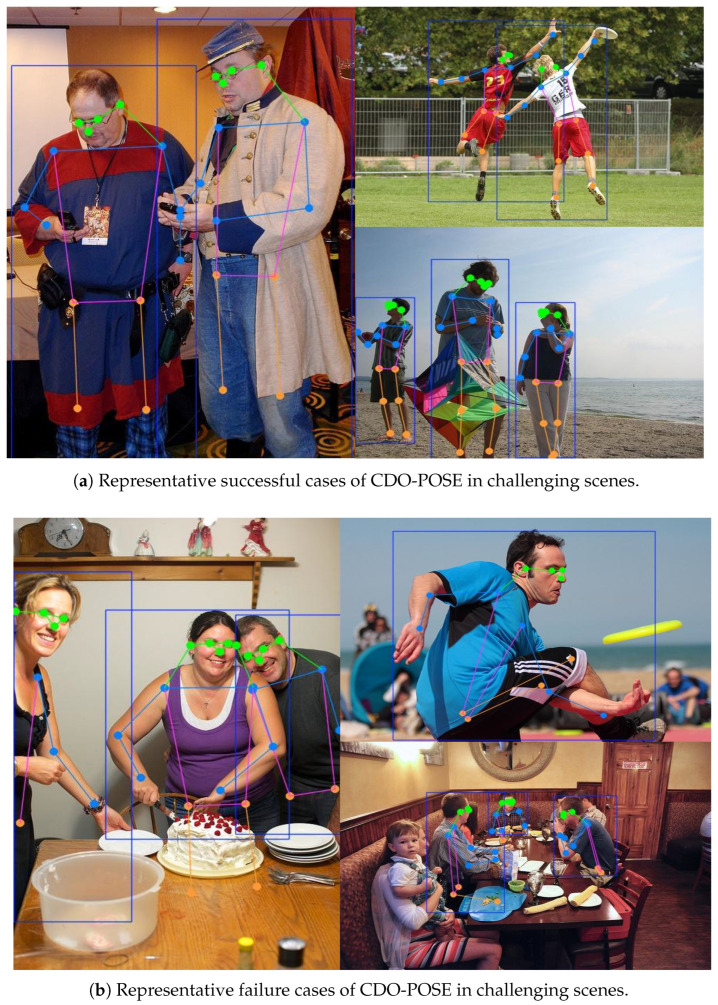
Qualitative results of CDO-POSE in challenging multi-person pose estimation scenarios [9].

Although CDO-POSE achieves favorable performance in common human pose estimation scenarios, our observations show that more obvious failure cases mainly arise in challenging scenes involving severe overlap between individuals and uncommon body poses. Representative qualitative results, including both successful and failure cases, are shown in Figure 10.

Specifically, substantial overlap between individuals increases the ambiguity of instance association, making it more difficult to distinguish body parts belonging to different subjects. In addition, uncommon poses may reduce the stability of pose estimation, as they deviate from more regular pose patterns. In such challenging cases, inaccurate keypoint localization is more likely to occur, particularly for occluded joints and distal keypoints, as illustrated by the representative failure examples in Figure 10b.

These results suggest that CDO-POSE still has room for improvement in complex multi-person scenes with rare pose configurations. Future work will focus on improving robustness in the presence of severe overlap between individuals and enhancing generalization to uncommon poses.

## 5. Conclusions

In this study, we propose a lightweight method based on YOLOv11 to balance parameters and accuracy for HPE tasks. We introduce the improved C3k2CAA to reduce the model’s parameter count and replace the upsample with the lightweight, efficient dynamic upsampling method DySample, which enhances model accuracy without increasing the number of parameters. Furthermore, we employ the OKS-Loss function to further improve accuracy. As a result, the model size is reduced to 6.7 M, while mAP@50 reaches 85.3 and mAP@50-95 reaches 57.0, which represents a 32.3% reduction in parameters compared to the baseline YOLOv11s, with only a 0.3 decrease in mAP@50-95. In addition, we successfully deployed CDO-POSE on the NVIDIA Jetson Orin Nano edge computing platform, where it achieved 39.79 FPS at 480p and 29.23 FPS at 720p. Moreover, its accuracy on the Jetson Orin Nano remains nearly identical to that on a high-performance NVIDIA 4090D GPU, confirming that CDO-POSE can maintain high precision and real-time inference even under limited computational resources. These results fully demonstrate the effectiveness and practicality of the proposed model for edge device deployment. 

## Figures and Tables

**Table 1:** Comparison of different methods on the **COCO2017 test-dev** set.

Method	Params (M)	mAP50	mAP75	mAP50-95	mAPM	mAPL
Large model						
Hourglass [49]	277.8	81.8	61.8	56.6	-	67.0
PETR [50]	220.5	90.4	79.6	-	68.1	79.0
ED-Pose [51]	218	92.3	80.9	72.7	67.6	80.8
SimpleBaseline [34]	68.6	91.2	80.1	71.6	68.7	77.2
Mask R-CNN [4]	63.0	87.3	68.7	63.1	57.8	71.4
**CDO-POSE**	6.7	85.3	62.1	57.0	51.0	67.1
Small model						
YOLO5s6_pose_ti_lite [47]	15.1	85.0	65.4	59.2	51.5	70.1
YOLOv8s-pose [38]	11.6	86.4	64.6	58.7	53.2	68.1
YOLOv11s-pose [52]	9.9	86.3	63.2	57.3	52.9	65.7
YOLOv5s_pose [47]	7.2	83.2	58.2	53.9	50.3	59.6
YOLOv7-tiny-pose [53]	6.1	80.3	47.3	45.8	45.9	47.1
**CDO-POSE**	6.7	85.3	62.1	57.0	51.0	67.1

**Table 2:** Comparison of different methods on the **CrowdPose test** set.

Method	Params (M)	mAP50	mAP75	mAP50-95	mAPM	mAPL
Large model						
PETR [50]	220.5	90.4	78.3	77.3	72.0	65.8
ED-Pose [51]	218	90.5	79.8	80.5	73.8	63.8
SimpleBaseline [34]	68.6	81.4	60.3	71.4	61.2	51.2
Mask R-CNN [4]	63.0	83.5	60.3	69.4	57.9	45.8
**CDO-POSE**	6.7	84.6	63.4	66.2	56.9	50.4
Small model						
YOLO5s6_pose_ti_lite [47]	15.1	81.3	54.1	50.7	50.7	43.1
YOLOv8s-pose [38]	11.6	85.8	66.0	68.3	61.7	51.6
YOLOv11s-pose [52]	9.9	85.9	65.0	67.7	61.1	50.8
YOLOv5s_pose [47]	7.2	78.2	49.4	46.7	46.7	39.7
YOLOv7-tiny-pose [53]	6.1	78.7	51.1	47.8	47.8	40.7
**CDO-POSE**	6.7	84.6	63.4	66.2	56.9	50.4

**Table 3:** Ablation results on the **COCO2017 test-dev** set.

C3k2CAA	DySample	OKS-Loss	Params (M)	mAP50	mAP75	mAP50-95	mAPM	mAPL
×	×	×	9.9	86.3	63.2	57.3	52.9	65.7
✓	×	×	6.7	85.2	60.1	54.9	50.3	63.5
×	✓	×	9.9	86.6	63.8	57.7	53.7	65.8
×	×	✓	9.9	86.6	66.0	59.8	54.6	69.2
✓	✓	×	6.7	85.4	60.7	55.5	50.9	64.2
×	✓	✓	9.9	86.9	65.9	59.9	54.6	69.3
✓	✓	✓	6.7	85.3	62.1	57.0	51.0	67.1

**Table 4:** Inference performance on Jetson Orin Nano.

Model	Params (M)	FPS (480p)	FPS (720p)
YOLOv8-pose	69.4	8.94	6.40
YOLOv11-pose	58.8	10.37	6.54
CDO-POSE	6.7	39.79	29.23

**Table 5:** Performance of **CDO-POSE** on **COCO2017 validation set** and **test-dev** across different devices.

Dataset	Devices	Model	Params (M)	GFLOPS	mAP50-95	mAP50	mAP75
COCO2017 Val	NVIDIA 4090D GPU	CDO-POSE	6.7	17.8	58.4	83.8	63.2
	Jetson Orin Nano	CDO-POSE	6.7	17.8	58.3	83.5	63.5
COCO2017 test-dev	NVIDIA 4090D GPU	CDO-POSE	6.7	17.8	57.0	85.3	62.1
	Jetson Orin Nano	CDO-POSE	6.7	17.8	56.9	84.9	62.1

## Data Availability

Data available in a publicly accessible repository.
